# Supplementary material for: Transferability of Human and Environmental Microbiome on Clothes as a Tool for Forensic Investigations
Source: Genes (Basel). 2024 Mar 19;15(3):375. doi: 10.3390/genes15030375 (PMC10970523; doi:10.3390/genes15030375)
Supplement: Supplementary file 1 [file genes-15-00375-s001.zip › genes-2902230-supplementary.pdf]

Transferability of human and environmental microbiome on clothes as a tool for forensic investigation

Supplementary Materials

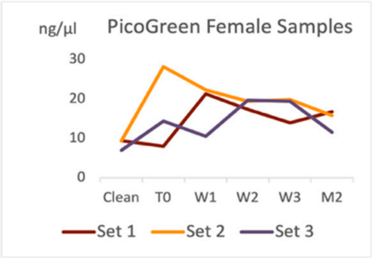

Supplementary Figure S1. A visual representation of the calculated concentrations from the Quant-iT™ PicoGreen™ measurements of the female samples collected after washing the t-shirt ("clean") and after wearing it at time 0, after 1,2 and 3 weeks, and after 2 months. Each of the three sets of replicates is reported with three different colours.

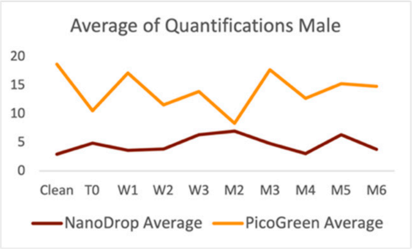

Supplementary Figure S2. Average concentrations of the male samples over time (T0-month 6), divided by quantification methods (NanoDrop™ vs Quant-iT™ PicoGreen™).

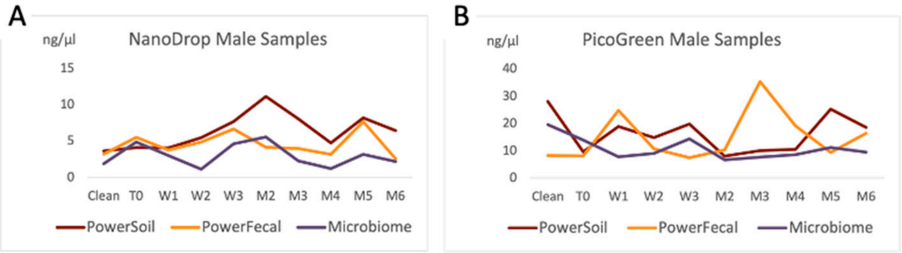

Supplementary Figure S3. Concentrations of the male samples over time (T0-month 6) measured with (a) NanoDrop™ and (B) Quant-iT™ PicoGreen™.

Supplementary Table S1. Core microbiomes identified in the study.

	Kingdom	Phylum	Class	Order	Family	Genus	Species	unique
**CORE MICROBIOME MALE SWABS**								
ASV1066	d__Bacteria	Firmicutes	Bacilli	Lactobacillales	Streptococcaceae	Streptococcus	Streptococcus Genus	06f825b512d903b9230e1a55d87359ee
ASV1088	d__Bacteria	Firmicutes	Bacilli	Staphylococcales	Staphylococcaceae	Staphylococcus	Staphylococcus Genus	65d43491988bfe557da4d86a5ba25dae
ASV1336	d__Bacteria	Actinobacteriota	Actinobacteria	Corynebacteriales	Corynebacteriaceae	Corynebacterium	Corynebacterium Genus	aa9b3a1418d146c262ec63305292065a
ASV1341	d__Bacteria	Actinobacteriota	Actinobacteria	Corynebacteriales	Corynebacteriaceae	Corynebacterium	Corynebacterium _aurimucosum	6a4c0e5943a7eb8c9f0b5b5e69171828
**CORE MICROBIOME T-SHIRTS POST WASHING**								
ASV1088	d__Bacteria	Firmicutes	Bacilli	Staphylococcales	Staphylococcaceae	Staphylococcus	Staphylococcus Genus	65d43491988bfe557da4d86a5ba25dae
ASV1336	d__Bacteria	Actinobacteriota	Actinobacteria	Corynebacteriales	Corynebacteriaceae	Corynebacterium	Corynebacterium Genus	aa9b3a1418d146c262ec63305292065a
ASV1341	d__Bacteria	Actinobacteriota	Actinobacteria	Corynebacteriales	Corynebacteriaceae	Corynebacterium	Corynebacterium _aurimucosum	6a4c0e5943a7eb8c9f0b5b5e69171828
ASV1623	d__Bacteria	Proteobacteria	Gammaproteo bacteria	Pseudomonadales	Moraxellaceae	Acinetobacter	Acinetobacter Genus	ea403646ed22d679fa4586263d8fc32f
**CORE MICROBIOME BOXES**								
ASV95	d__Bacteria	Proteobacteria	Alphaproteob acteria	Rhizobiales	Beijerinckiaceae	Methylobacteriu m-Methylorubrum	Methylobacteriu m_soli	310c4295d080f79e09b15ee634bf65cf
ASV103	d__Bacteria	Proteobacteria	Alphaproteob acteria	Rhizobiales	Beijerinckiaceae	Methylobacteriu m-Methylorubrum	Methylobacteriu m-Methylorubrum Genus	e0f50c5adf537a0a3a63e61720b38ed5
ASV1017	d__Bacteria	Firmicutes	Bacilli	Paenibacillales	Paenibacillaceae	Paenibacillus	Paenibacillus Genus	ba6c32d2cb692cd9e0fdc3693a3996e5
ASV1019	d__Bacteria	Firmicutes	Bacilli	Paenibacillales	Paenibacillaceae	Paenibacillus	Paenibacillus_ti monensis	2c16378eb83fb581fa19dc7850ef14e4
ASV1022	d__Bacteria	Firmicutes	Bacilli	Paenibacillales	Paenibacillaceae	Paenibacillus	Paenibacillus Genus	d3fcc258e178740b8efd9946f7f08cd3
ASV1028	d__Bacteria	Firmicutes	Bacilli	Bacillales	Bacillaceae	Bacillus	Bacillus Genus	b8c545c99dea06d010eb0bf2c6572d1c
ASV1033	d__Bacteria	Firmicutes	Bacilli	Bacillales	Planococcaceae	Lysinibacillus	Lysinibacillus Genus	1fb6ab5be1d427ef2ed5faeaacd60eca
ASV1219	d__Bacteria	Actinobacteriota	Actinobacteria	Propionibacteriales	Nocardioidaceae	Nocardioides	Nocardioides Genus	7eb6d17edc700607f19d055c57c86222
**CORE MICROBIOME TRANSFER AT T14 DAYS**								
ASV1066	d__Bacteria	Firmicutes	Bacilli	Lactobacillales	Streptococcaceae	Streptococcus	Streptococcus Genus	06f825b512d903b9230e1a55d87359ee
ASV1678	d__Bacteria	Proteobacteria	Gammaproteo bacteria	Burkholderiales	Neisseriaceae	Neisseria	Neisseria Genus	f1860fe716257bd5fd5c4c6a16cf3b95
**CORE MICROBIOME TRANSFER AT T21 DAYS**								
ASV1266	d__Bacteria	Actinobacteriota	Actinobacteria	Micrococcales	Micrococcaceae	Micrococcus	Micrococcus Genus	0c579d21280801f02a641e1608606927
**CORE MICROBIOME TRANSFER AT T60 DAYS**								
ASV1066	d__Bacteria	Firmicutes	Bacilli	Lactobacillales	Streptococcaceae	Streptococcus	Streptococcus Genus	06f825b512d903b9230e1a55d87359ee
ASV1266	d__Bacteria	Actinobacteriota	Actinobacteria	Micrococcales	Micrococcaceae	Micrococcus	Micrococcus Genus	0c579d21280801f02a641e1608606927
**CORE MICROBIOME TRANSFER AT T90 DAYS**								
ASV1066	d__Bacteria	Firmicutes	Bacilli	Lactobacillales	Streptococcaceae	Streptococcus	Streptococcus Genus	06f825b512d903b9230e1a55d87359ee
ASV1266	d__Bacteria	Actinobacteriota	Actinobacteria	Micrococcales	Micrococcaceae	Micrococcus	Micrococcus Genus	0c579d21280801f02a641e1608606927
**CORE MICROBIOME TRANSFER AT T120 DAYS**								

ASV1266	d__Bacteria	Actinobacteriota	Actinobacteria	Micrococcales	Micrococcaceae	Micrococcus	Micrococcus Genus	0c579d21280 801f02a641e1 608606927
**CORE MICROBIOME TRANSFER AT T150 DAYS**								
ASV1066	d__Bacteria	Firmicutes	Bacilli	Lactobacillales	Streptococcaceae	Streptococcus	Streptococcus Genus	06f825b512d9 03b9230e1a5 5d87359ee
ASV1266	d__Bacteria	Actinobacteriota	Actinobacteria	Micrococcales	Micrococcaceae	Micrococcus	Micrococcus Genus	0c579d21280 801f02a641e1 608606927
**CORE MICROBIOME TRANSFER AT T180 DAYS**								
ASV1066	d__Bacteria	Firmicutes	Bacilli	Lactobacillales	Streptococcaceae	Streptococcus	Streptococcus Genus	06f825b512d9 03b9230e1a5 5d87359ee
ASV1266	d__Bacteria	Actinobacteriota	Actinobacteria	Micrococcales	Micrococcaceae	Micrococcus	Micrococcus Genus	0c579d21280 801f02a641e1 608606927
